# Revisiting Mac-2-Binding Protein Glycosylation Isomer (M2BPGi) for Diagnosing High-Risk Liver Fibrosis in Chronic Hepatitis B Patients: A Stepwise Diagnostic Analysis

**DOI:** 10.12688/f1000research.147153.2

**Published:** 2024-07-12

**Authors:** Muhammad Begawan Bestari, Haryono Haryono, Muhammad Palar Wijaya, Dolvy Girawan, Nenny Agustanti, Eka Surya Nugraha

**Affiliations:** 1 Internal Medicine, Padjadjaran University, Bandung, West Java, 40161, Indonesia

**Keywords:** M2BPGi, chronic hepatitis B, fibrosis, diagnostic, APRI, FIB-4, AAR

## Abstract

**Background:**

The level of liver fibrosis is the basis for the treatment of chronic hepatitis B (CHB), and it is necessary to adapt non-invasive liver fibrosis modalities. We aimed to investigate the use of M2BPGi as a single or combined diagnostic modality for liver fibrosis in CHB patients through a stepwise diagnostic analysis.

**Methods:**

Cross-sectional data were taken from patients between October 2021 and August 2022. Demographic data, blood profile, liver function, and liver stiffness were measured in CHB patients over 18 years old, willing to take part in the research, and had complete data. APRI, FIB-4, and AAR were calculated using the well-known formulas. Serum M2BPGi-levels were converted into a cut-off index (COI). The patients were divided into low-risk (LR) and high-risk fibrosis (HR) groups. A cut-off for each predictor variable to differentiate between the LR and HR groups was determined. The obtained cut-off was assessed for its association with the grouping of liver elastography results. Models to diagnose the liver stiffness measurement (LSM) ≥8 kPa were created and compared through multivariate and ROC analyses.

**Results:**

The number of patients that met the inclusion and exclusion criteria was 143 (HR = 65, LR = 78). The cut-off for diagnosing LSM ≥8kPa was 0.311, 0.742, 0.635, and 1.434 for APRI, FIB-4, AAR, and M2BPGi, respectively. This cut-off was significantly associated with the results of the HR and LR groupings. A multivariate analysis found that FIB4, AAR, and M2BPGi added significantly to the model. Statistically, the most optimal use of M2BPGi was combined with FIB-4, with an AUC of 0.835.

**Conclusions:**

The optimal cut-off of M2BPGi for diagnosing high-risk liver fibrosis in this study was 1.434. M2BPGi should be used with FIB-4 as a diagnostic tool for diagnosing liver fibrosis, especially in the absence of a liver biopsy or elastography.

## Introduction

Globally, an estimated 296 million people, with 18 million in Southeast Asia, are projected to have a CHB infection by the World Health Organization (WHO). The annual rate of new infections is about 1.5 million. Hepatocellular carcinoma (HCC) and liver fibrosis caused by hepatitis B were responsible for 820,000 deaths in 2019. ^1^ In determining the severity of fibrosis or inflammation in the liver, a liver biopsy is the primary option, but it is an invasive procedure. The American Association for the Study of Liver Diseases (AASLD) has suggested several non-invasive techniques. ^2^

Most non-invasive techniques were easily performed and reproduced in the outpatient clinic, have low cost, and have been validated for specific populations were the other advantages. ^3^

Mac-2-binding protein (M2BP) is a glycoprotein that, when changes are made to its N-glycan residue, forms M2BPGi. M2BPGi is produced by hepatic stellate cells (HSCs) and it induces profibrotic cytokine expression in Kupffer cells (KCs), namely Mac-2. Subsequently, Mac-2 activates HSCs and cause fibrogenesis. ^4^^,^^5^ M2BPGi has been widely used to predict liver fibrosis and cirrhosis in different chronic liver diseases. ^5^^–^^18^ In several previous studies, M2BPGi helped to diagnose liver fibrosis in a CHB population ^6^^,^^11^ and it could be used as a single predictor variable to diagnose liver fibrosis grade. ^12^^,^^13^ This marker could also complement and be used with other modalities. ^14^^,^^15^ The most accurate non-invasive methods to assess fibrosis are liver stiffness measurements (elastography), followed by several scoring methods such as the AST-to-platelet ratio index (APRI), the fibrosis index based on four factors (FIB-4), and the AST-to-ALT ratio (AAR). ^2^^,^^19^^–^^21^

Adapting non-invasive liver fibrosis modalities to each type of chronic liver disease and each region is necessary due to the heterogeneity of outcomes. A stepwise diagnostic analysis has yet to be conducted to determine whether M2BPGi should be utilized alone or in conjunction with modalities to assess liver fibrosis. Thus, we aimed to investigate the use of M2BPGi as a single or combined diagnostic modality for liver fibrosis in CHB patients through a stepwise diagnostic analysis.

## Methods

### Study design and patients

We obtained ethical approval from the Research Ethics Committee of Dr. Hasan Sadikin General Hospital Bandung (LB.02.01/X.6.5/299/2021) in order to protect the rights and welfare of research subjects, and to guarantee the study to be conducted according to ethical, legal, social implications, and other applicable regulations. This was a cross-sectional study; the subjects of this study were patients from the Gastroenterohepatology outpatient clinic, Hasan Sadikin General Hospital, Indonesia, between October 2021 and August 2022. All patients were older than 18 years and were positive for serum hepatitis B surface antigen (HBsAg) for at least six months. The criteria for exclusion were as follows: 1) acute hepatitis; 2) acute exacerbation of chronic hepatitis; 3) hepatitis C; 4) autoimmune liver disease; 5) hepatitis B co-infection with hepatitis C or HIV; 6) co-morbidities (type 2 diabetes mellitus, heart disease, chronic kidney disease, pulmonary tuberculosis, or cancer); 7) patients with a history of alcohol use (>20 grams of alcohol per day); 8) pregnant or breastfeeding woman; 9) body mass index (BMI) >27 kg/m ^2^; 10) hemoglobin <5 g/dL; and 11) pulmonary fibrosis, chronic pancreatitis, liver cancer, or pancreatic cancer.

There are several ways to determine the optimum sample size for a binary logistic regression analysis. First, using the rule of thumb method with N = number of independent variables multiplied by 10-50, the value for our sample size was between 30 and 150. Another method is by including the prevalence correction factor with the formula: N = 10 k/p, where k is the number of independent variables and p is the prevalence correction factor. ^22^ In our study subjects, the prevalence was 45%; thus, the number of efficient samples is 66.67 = 67 patients. Our study was conducted on 143 subjects.

### Clinical data and laboratory test

CHB patients who met the inclusion and exclusion criteria received information about the study. After obtaining written (informed) consent, their demographic data were collected. The research subjects underwent supporting examinations of liver elastography and routine laboratory investigations, including measurements of CBC, AST, ALT, PT, INR, and M2BPGi serum levels. All laboratory examinations were carried out in the clinical pathology laboratory of Hasan Sadikin General Hospital. All equipment underwent quality control before use and was used by trained technicians. The formulas used to calculate the non-invasive liver fibrosis scores are as follows ^23^^,^^24^:

$$\text{APRI}=\frac{\left[\mathrm{AST}(U/L)/\mathrm{ULN}\times 100\right]}{\text{Platelet}(\times 10^{9}/L)}$$

$$\mathrm{FIB}-4=\frac{\left[\text{age}(\text{years})\times \text{AST}(U/L)\right]}{\left[\text{Platelet}(\times 10^{9}/L)\times \sqrt{\text{ALT}(U/L)}\right]}$$

$$\text{AAR}=\frac{\text{AST}}{\mathrm{ALT}}$$

### Transient elastography

Transient elastography (TE) is a non-invasive examination to determine the level of liver fibrosis, with results given in the form of an LSM (in kPa). This examination was performed using the FibroScan ^®^ 502 Series F00734 (Echosens, Paris, France) with the M or XL probe. Liver stiffness was expressed as the median value of more than ten valid examinations. The value of LSM can be trusted if the success rate is greater than 60% and the interquartile range (IQR) ratio to the median liver stiffness is below 30%. This examination was performed by a gastroenterohepatologist at Hasan Sadikin General Hospital. FibroScan was cleaned and disinfected regularly to ensure optimal operation. Delegates from Echosens calibrate FibroScan yearly.

### M2BPGi measurements

Serum M2BPGi levels were measured using the HISCL M2BPGi reagent kit (Sysmex, Hyogo, Japan, Catalogue number: CB090850) (Supplier: PT. Saba Indomedika) and an automatic immune analyzer HISCL 800 (Sysmex, Hyogo). In total, from the M2BPGi reagent kit, 50 μL of R1 reagent, 30 μL of R2 reagent, 600-4200 μL of washing solution, 100 μL of R3 reagent, 50 μL of R4 reagent, and 100 μL of R5 reagent, were used. The results of the M2BPGi serum level were expressed as a cut-off index (COI). The COI was calculated using the following formula ^25^:

$$\mathrm{COI}=\frac{\left([M2\text{BPGi}]\;\text{sample}–[M2\text{BPGi}]\;\text{negative control}\right)}{\left([M2\text{BPGi}]\;\text{positive control}\;[M2\text{BPGi}]\;\text{negative control}\right)}$$

### Statistical analysis

First, a normality test was conducted to determine the subsequent statistical analysis procedure. The patient characteristic data are presented in Table 1; those with a normal distribution are expressed as the mean and standard deviation, while those that are not normally distributed are presented as the median and minimum–maximum values. The results of the transient elastography were used to classify the subjects into two groups: high-risk fibrosis (HR) (LSM ≥8 kPa) and low-risk fibrosis (LR) (LSM < 8 kPa). ^3^

**Table 1.** Baseline characteristics of 143 study participants.

Characteristic	Value
Total number of patients (n)	n = 143
Age (years) ^†^	42 (20-76)
Sex (male/female) *	77/66
AST (IU/L) ^†^	26 (12-143)
ALT (IU/L) ^†^	35 (9-279)
Platelet (×10 ^9^/L) ^‡^	237.2 (±79.25)
M2BPGi (COI) ^†^	1.04 (0.22-20)
APRI ^†^	0.31 (0.1-2.70)
FIB-4 ^†^	0.75 (0.19-8.43)
AAR ^†^	0.73 (0.31-1.88)
Liver elastography (kPa) ^†^	7.4 (2.5-70.6)

AST: aspartate aminotransferase; ALT: alanine aminotransferase; M2BPGi: Mac-2-binding protein glycosylation isomer; APRI: AST-to-platelet ratio index; FIB4: fibrosis index based on 4 factors; AAR: AST-to-ALT ratio.

* Value is given as a proportion.

^†^ Values are the medians with ranges in parentheses.

^‡^ Values are the means with standard deviations in parentheses.

Each predictor variable underwent an ROC analysis using SPSS version 20 (IBM Corp. 2011. Armonk, NY, USA, RRID:SCR_00286) to develop cut-off values based on Youden’s index to balance sensitivity and specificity in diagnosing high-risk fibrosis. STATA 17 (StataCorp. 2021. Stata Statistical Software: Release 17. College Station, TX: StataCorp LLC; RRID: SCR_012763) was used for the stepwise diagnostic analysis. Each predictor variable was grouped based on their cut-off value. The association between each grouped predictor variable and the HR and LR groupings was assessed using a chi-square analysis. Diagnostic models were created and subjected to stepwise logistic regression. The receiver operating characteristic (ROC) analysis determined each model’s accuracy. Then, ROC analysis comparisons were carried out between each model to evaluate whether there was a significant difference in adding predictors. A two-tailed p < 0.05 was considered statistically significant.

## Results

### Subject characteristics

**Figure 1.**
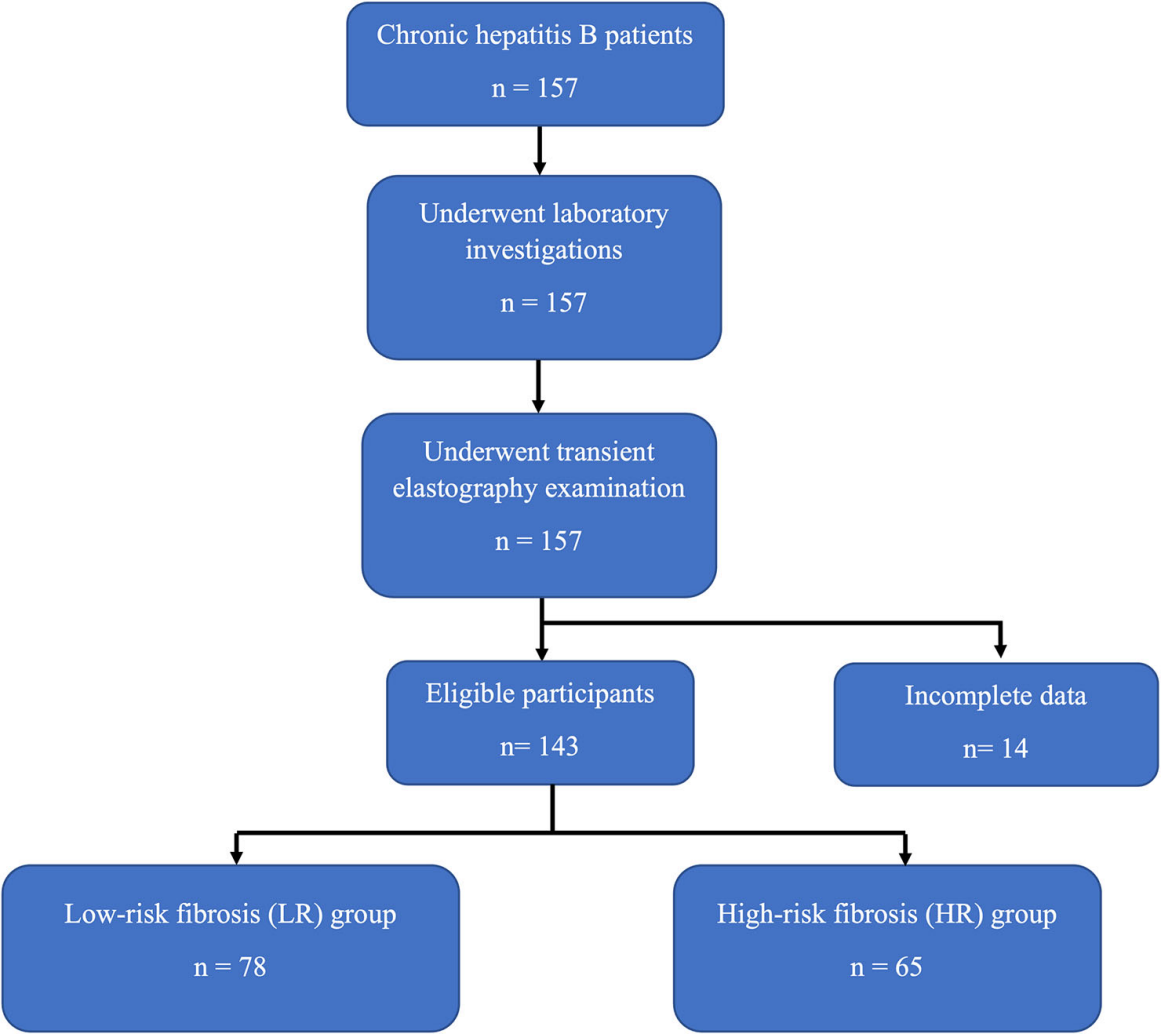
Flowchart for selection of study subjects.

The total number of CHB patients at Hasan Sadikin General Hospital during the research period was 157. All patients were entered into the Hasan Sadikin Chronic Hepatitis B Registry. After all examinations and data collection, 14 patients were excluded because of incomplete data. The number of patients who met the inclusion and exclusion criteria was 143. The patients’ baseline characteristics are summarized in Table 1. Based on the transient elastography results, the patients were split into two groups: high-risk fibrosis (HR) (n = 65) and low-risk fibrosis (LR) (n = 78) groups. The flowchart for the selection of the participants is shown in Figure 1.

### Bivariate analysis for high-risk fibrosis

Each predictor underwent an ROC analysis to obtain the optimal cut-off point. It was found that the best cutoff values for M2BPGi, APRI, FIB-4, and AAR were 1.434, 0.311, 0.742, and 0.635, respectively. The results of the bivariate analysis between the predictor variables for the HR and LR groups are shown in Table 2. Based on the liver elastography results, M2BPGi, APRI, FIB-4, and AAR were significantly associated with the HR and LR groupings.

**Table 2.** Bivariate analysis for high-risk fibrosis (LSM ≥8 kPa) amongst 143 patients.

Variable	LSM < 8 (n = 78)	LSM ≥ 8 (n = 65)	p-value
APRI <0.311	53 (68%)	18 (28%)	<0.001
APRI ≥0.311	25 (32%)	47 (72%)	
FIB4 <0.742	57 (73%)	12 (18%)	<0.001
FIB4 ≥0.742	21 (27%)	53 (82%)	
AAR <0.635	33 (42%)	12 (18%)	0.002
AAR ≥0.635	45 (58%)	53 (82%)	
M2BPGi <1.434	70 (90%)	28 (43%)	<0.001
M2BPGi ≥1.434	8 (10%)	37 (57%)	

* p-value from the chi-square test. LSM: liver stiffness measurement; M2BPGi Mac-2-binding protein glycosylation isomer; APRI: AST-to-platelet ratio index; FIB4: fibrosis index based on 4 factors; AAR: AST-to-ALT ratio.

### Stepwise multivariate analysis for high-risk fibrosis

Through a Spearman analysis, APRI with FIB-4 had the strongest correlation, with a correlation coefficient of 0.78. Based on the correlation coefficient data, it was decided to exclude APRI from the diagnostic model to avoid violating the multicollinearity rule. The models used different combinations of M2BPGi, FIB-4, and AAR. Model 1 consisted of M2BPGi only, followed by the addition of other predictors one by one to produce Model 2, which consisted of M2BPGi and AAR; Model 3, which consisted of M2BPGi and FIB-4; and Model 4, which consisted of M2BPGi, FIB-4, and AAR. All variables were analyzed to assess the association. The results of the multivariate analysis are shown in Table 3. All models were statistically significant (p-value < 0.001) compared to the intercept-only model and had a Nagelkerke R ^2^ fit at 0.31, 0.36, 0.46, and 0.49 for Models 1, 2, 3, and 4, respectively.

**Table 3.** Multivariate analysis for predictors of HR group (LSM ≥8kPa) amongst 143 patients.

	Predictor variable	Adjusted OR	95% CI	p-value
Model 1	M2BPGi ≥1.434	11.562	4.791-27.903	<0.001
Model 2	M2BPGi ≥1.434	11.424	4.609-28.316	<0.001
	AAR ≥0.635	3.162	1.3-7.6	0.011
Model 3	M2BPGi ≥1.434	6.297	2.409-16.461	<0.001
	FIB-4 ≥0.742	7.44	3.161-17.511	<0.001
Model 4	M2BPGi ≥1.434	6.476	2.389-17.558	<0.001
	FIB-4 ≥0.742	6.873	2.882-16.388	<0.001
	AAR ≥0.635	2.685	1.048-6.88	0.04

The p-value is the result of the binary logistic regression analysis. M2BPGi: Mac-2-binding protein glycosylation isomer; APRI: AST-to-platelet ratio index; FIB4: fibrosis index based on 4 factors; AAR: AST-to-ALT ratio.

### Model’s accuracy for diagnosing high-risk fibrosis

The ROC analysis’s area under the curve (AUC) evaluates each model’s accuracy ( Table 4). The model utilizing M2BPGi with the best accuracy was in combination with FIB-4 and AAR. The abilities of each model were compared, and the results are shown in Table 5.

**Table 4.** ROC analysis of the models for diagnosing high-risk fibrosis (LSM ≥8 kPa) amongst 143 patients.

Model	AUC	95% CI
Model 1	0.733	0.664-0.803
Model 2	0.782	0.71-0.854
Model 3	0.835	0.77-0.9
Model 4	0.852	0.788-0.916

AUC: area under curve; Model 1: M2BPGi; Model 2: M2BPGi and AAR; Model 3: M2BPGi and FIB-4; Model 4: M2BPGI, FIB4, and AAR; M2BPGi: Mac-2-binding protein glycosylation isomer; APRI: AST-to-platelet ratio index; FIB4: fibrosis index based on 4 factors; AAR: AST-to-ALT ratio.

**Table 5.** ROC comparison of the models for diagnosing high-risk fibrosis (LSM ≥8 kPa) amongst 143 patients.

Model	AUC difference	p-value
Model 1 vs. Model 2 vs. Model 3 vs. Model 4	overall differences	0.0019 *
Model 1 vs. Model 2	4.9% (post hoc)	0.017 *
Model 1 vs. Model 3	10.2% (post hoc)	0.0007 *
Model 1 vs. Model 4	11.9% (post hoc)	0.0001 *
Model 2 vs. Model 3	5.3% (post hoc)	0.1013
Model 2 vs. Model 4	7% (post hoc)	0.01 *
Model 3 vs. Model 4	1.7% (post hoc)	0.1239

The p-value is the result of the ROC comparison.

Model 1: M2BPGi; Model 2: M2BPGi and AAR; Model 3: M2BPGi and FIB-4; Model 4: M2BPGI, FIB4, and AAR; M2BPGi: Mac-2-binding protein glycosylation isomer; APRI: AST-to-platelet ratio index; FIB4: fibrosis index based on 4 factors; AAR: AST-to-ALT ratio.

* Statistically significant difference.

## Discussion

Non-invasive methods for assessing liver fibrosis are currently being developed. It has been demonstrated that M2BPGi is a good biomarker for evaluating liver fibrosis. ^5^^–^^18^ A stepwise diagnostic analysis has not yet been conducted to determine the value of M2BPGi in assessing liver fibrosis. Here, we aimed to investigate the use of M2BPGi as a single or combined diagnostic modality for liver fibrosis in CHB patients through a stepwise diagnostic analysis.

Currently, a liver biopsy is the gold standard for assessing liver inflammation and fibrosis. However, biopsy is an invasive procedure and has several risks, such as bleeding, hematoma, and mild discomfort to severe pain; hence, it is not suitable for routine use. ^26^ There are various non-invasive methods to assess liver fibrosis. Liver elastography is the primary alternative for assessing liver fibrosis. To determine liver stiffness in this study, we used the FibroScan ^®^ tool and determined the fibrosis class based on the EASL recommendations in FibroScan ^®^. In the outside liver clinic settings, the results of LSM are divided into ≥8 kPa for high-risk fibrosis and <8 kPa for low-risk. ^3^ Operator skills and experience, the selection of appropriate probes, and special conditions such as obesity are challenges in applying the liver elastography method. There was no significant difference in gender proportion between the HR and LR groupings. However, the HR group had an increment in age compared with the LR group.

The AUC of M2BPGi, APRI, FIB-4, and AAR were 0.721 (95%CI 0.631-0.811), 0.757 (95%CI 0.678-0.836), 0.822 (95%CI 0.753-0.891), 0.618 (95%CI 0.526-0.71), respectively in our samples. The ROC coordinate points obtained a new cut-off for M2BPGi, APRI, FIB-4, and AAR ( Table 2). This optimal cut-off is not too different from those of Zou’s study ^15^ for diagnosing METAVIR grade ≥F2 with an APRI cut-off of 0.51, a FIB-4 cut-off of 0.92, and an AAR cut-off of 0.55. In a study to distinguish LSM ≥7 kPa in Vietnam, cut-offs of 0.5 and 1.8 were obtained for APRI and FIB-4, respectively. ^12^ In patients with liver elastography results ≥9 kPa in Egypt, the cut-offs were at 0.256, 0.74, and 0.8 for APRI, FIB-4, and AAR, respectively. ^27^ For predicting Knodell histologic activity index (HAI) ≥F2 results, APRI and FIB-4 had the best cut-offs at 0.9 and 0.35, respectively. ^11^ The APRI cut-offs found to date vary widely. In diagnosing LSM ≥8 kPa, the value appeared lower in this study (0.311) compared to other studies, regardless of the dependent variable groupings. ^11^^,^^12^^,^^15^ From the various reviews that have been done, the possibility of this result could occur because the baseline data on platelet count in our population was considered to be the highest, with an average of 237.2 (×10 ^9^/L). A high platelet count causes the distribution of APRI values to be lower, lowering the further obtained cut-off. Zou, ^15^ Jekarl, ^11^ and Bui ^12^ had median platelet counts of 155, 185, and 206, respectively, resulting in a decreasing trend of APRI cut-offs of 1.06, 0.9, and 0.569, respectively.

The research regarding the ability of non-invasive liver fibrosis modalities can be broadly divided into two areas, using liver biopsy or TE as the comparison. We are among those who used TE results as the gold standard. While TE was rarely used, Bui et al. found an M2BPGi cut-off of 0.79 for diagnosing LSM ≥7 kPa. ^12^ The cut-off of M2BPGi that we obtained to diagnose LSM ≥8 kPa was 1.434. M2BPGi levels were higher in women in this study (p-value = 0.022). Our cut-off is quite close to the previous cut-off for diagnosing significant liver fibrosis using biopsy as the gold standard by Yeh et al. ^14^ and Ishii et al. ^6^ at 1.345 and 1.4, respectively.

Our M2BPGi cut-off is greater than 1, which we suspect is due to aging. There was a significant difference between the ages of the HR and LR groups in our study, with a weak correlation between age and M2BPGi levels (r = 0.314). In several previous studies, the cut-off for M2BPGi was around COI 1. ^6^^,^^10^^,^^11^^,^^13^^,^^15^ Cheng et al. found that aging increases M2BPGi levels in healthy patients. ^18^ This finding may explain why our cut-off results were more than one. However, the effects of aging on M2BPGi levels require further research. Based on the cut-off found, the four predictor variables were divided into categorical data; all predictors were associated with the categorical classification of liver elastography with a cut-off of 8 kPa ( Table 2).

The highest bivariate correlation analysis results were found between APRI and FIB-4; this was based on the fact that both indices consist of AST and platelet counts as the primary variables. This robust correlation made APRI excluded to produce an unbiased multivariate analysis. APRI was also excluded because its association with HR and LR grouping was weaker than FIB-4 ( Table 2). Four models were developed involving M2BPGi, FIB-4, and AAR to assess the performance of M2BPGi on its own. M2BPGi, FIB-4, and AAR (Model 4) were able to predict the HR group. Patients with any result equal to or more than the M2BPGi, FIB-4, and AAR cut-offs will result in a probability of 6.476, 6.873, and 2.685, respectively, for classification into the HR group. If used alone, each COI M2BPGi value ≥1.434 will produce a probability of 11.562 ( Table 3).

Model 4 had the best diagnostic ability with an AUC of 0.852 ( Table 4). The use of M2BPGi as a single modality (Model 1) in diagnosing high-risk liver fibrosis was quite good, with an AUC of 0.733. In diagnosing liver biopsy at ≥F2, Yeh et al. ^14^ and Zou et al. ^15^ obtained an AUC for M2BPGi of 0.78 and 0.753, respectively. Bui et al. found an AUC of 0.77 for diagnosing LSM ≥7 kPa. ^12^ Ichikawa et al., in determining F≥2 based on the revised Inuyama classification, found that M2BPGi had an AUC of 0.713. ^10^ In the group of patients with treatment-naïve CHB to diagnose portal fibrosis without septal involvement (F≥2), an AUC of 0.77 was obtained by Ishii et al. ^6^

There were significant differences in the diagnostic abilities of the models (p = 0.0019); a post hoc analysis was performed to determine whether the addition of a modality was statistically significant ( Table 5). In Model 2, M2BPGi was coupled with AAR, which increased the diagnostic capability compared to the M2BPGi-only model by around 4.9%. Model 3, which consisted of M2BPGi and FIB-4, was statistically the best model, with an AUC of 0.835. The addition of FIB-4 increased the AUC to 10.2%. Adding AAR to Model 3 to form Model 4 increased the diagnostic capability by 1.7% but this was not statistically significant. Therefore, Model 3 was the most efficient diagnostic model. M2BPGi can be used efficiently, and its application should be combined with FIB-4 to diagnose high-risk liver fibrosis.

In some earlier studies, the use of M2BPGi combined with other variables was proposed, as was performed by Yeh et al. ^14^ and Zou et al. ^15^ Yeh et al. supported using M2BPGi in models involving age and platelet counts to increase the specificity in the prediction of advanced fibrosis. ^14^ Zou et al. suggested measuring M2BPGi levels as a complementary method for liver biopsies and elastography. ^15^ However, both studies showed that the AUC value of M2BPGi was always superior to other scoring methods. ^14^^,^^15^ Bui et al. found that M2BPGi and APRI had the same AUC value (0.77) as a single indicator. However, in combining M2BPGi with other modalities, they only formed a single model to predict significant fibrosis using M2BPGi and APRI. By adding APRI to M2BPGi, the accuracy in detecting LSM ≥7 kPa was increased. Based on the high coefficient correlation between M2BPGi and liver elastography results, the paper stated that M2BPGi could be used as an alternative liver fibrosis test in CHB patients, especially in settings with limited resources. ^12^ Mak et al. performed an ROC analysis and created two predictive models for F3/F4 biopsy results. M2BPGi always produced statistically significant correlation in both models, while APRI, FIB-4, and AAR did not. They stated that M2BPGi was a potential marker for easily diagnosing F3/F4 without the need for a liver biopsy. ^13^

This study of serum M2BPGi levels in CHB patients aimed to aid its diagnostic application outside of liver clinic settings. The use of M2BPGi levels as part of a non-invasive method for diagnosing liver fibrosis outcomes based on liver elastography values was compared with several scoring methods. In our study, M2BPGi showed good diagnostic performance when used alone. However, our stepwise diagnostic analysis found that M2BPGi had a better result in diagnosing liver fibrosis when combined with FIB-4. The findings of COI M2BPGi ​​≥1.434 and FIB-4 ≥0.742 had an AUC of 85.2% in diagnosing high-risk liver fibrosis conditions. If those data ​​are obtained simultaneously, further liver fibrosis investigations should be done.

Our study was conducted cross-sectionally; the causal relationships were actually limited to associations. Potential bias has been avoided. To avoid selection bias, research participants comprehensively excluded various conditions affecting the purity of research samples and the examination results (TE and M2BPGi). We tried to include all patients initially because of the basis for establishing the Hepatitis B registry, but a majority reduced the number due to the completeness of the data. All data was obtained from objective sources (letters) to avoid information bias. The laboratory analyst did not know the patient participation status in the research. The patient's diagnosis was also unknown to the TE operator.

Since this is the first comprehensive statistical analysis performed on M2BPGi utilization, future studies should examine the use of serum M2BPGi levels by applying the stepwise diagnostic analysis method. In addition, validating research using model findings from our research can be carried out in other chronic liver diseases. In conclusion, after considering all the statistical comparisons and the stepwise diagnostic analysis, we believe that M2BPGi should be used with FIB-4 as a diagnostic tool for liver fibrosis, especially in the absence of liver biopsies or elastography.

### Ethical considerations

The Research Ethics Committee of Dr. Hasan Sadikin General Hospital Bandung granted permission on 20 October 2021 to conduct this research (LB.02.01/X.6.5/299/2021). After obtaining written (informed) consent, their demographic data were collected.

## Data Availability

Figshare: CHB for stepwise M2BPGi – Rev 1, https://doi.org/10.6084/m9.figshare.26195954.v1. ^28^ This project contains the following underlying data:
• CHB for stepwise M2BPGi.sav (the raw data for the study) Data are available under the terms of the Creative Commons Attribution 4.0 International license (CC-BY 4.0). Figshare: Participant information sheets, https://doi.org/10.6084/m9.figshare.25383106.v1. ^29^ Data are available under the terms of the Creative Commons Attribution 4.0 International license (CC-BY 4.0). Figshare: Informed consent form for sample, https://doi.org/10.6084/m9.figshare.25383157.v1. ^30^ Data are available under the terms of the Creative Commons Attribution 4.0 International license (CC-BY 4.0). Figshare: Reproducibility Checklist - Stepwise Diagnostic Analysis, https://doi.org/10.6084/m9.figshare.26195978.v1. ^31^ Data are available under the terms of the Creative Commons Attribution 4.0 International license (CC-BY 4.0). STARD checklist for ‘Revisiting Mac-2-Binding Protein Glycosylation Isomer (M2BPGi) for Diagnosing High-Risk Liver Fibrosis in Chronic Hepatitis B Patients: A Stepwise Diagnostic Analysis’. https://doi.org/10.6084/m9.figshare.26195972.v1
