# Supplementary material for: Revisiting Mac-2-Binding Protein Glycosylation Isomer (M2BPGi) for Diagnosing High-Risk Liver Fibrosis in Chronic Hepatitis B Patients: A Stepwise Diagnostic Analysis
Source: F1000Res. 2024 Jul 12;13:280. Originally published 2024 Apr 16. [Version 2] doi: 10.12688/f1000research.147153.2 (PMC11294808; doi:10.12688/f1000research.147153.2)
Supplement: Peer review history

# Reviewer report: Reviewer response for version 2

**DOI:** 10.5256/f1000research.168987.r302387

**Published:** 2024-08-01

**Authors:** Kemal Fariz Kalista

**Affiliations:** 1 Universitas Indonesia, Depok, West Java, Indonesia; 2 Internal Medicine, Cipto Mangunkusumo Hospital, Jakarta, Jakarta, Indonesia

The authors did a great job, previous comment was replied very appropiately. I think no more further comment.

Is the work clearly and accurately presented and does it cite the current literature?

Yes

If applicable, is the statistical analysis and its interpretation appropriate?

Yes

Are all the source data underlying the results available to ensure full reproducibility?

Yes

Is the study design appropriate and is the work technically sound?

Yes

Are the conclusions drawn adequately supported by the results?

Partly

Are sufficient details of methods and analysis provided to allow replication by others?

Yes

Reviewer Expertise:

Hepatology

I confirm that I have read this submission and believe that I have an appropriate level of expertise to confirm that it is of an acceptable scientific standard.

# Reviewer report: Reviewer response for version 2

**DOI:** 10.5256/f1000research.168987.r302388

**Published:** 2024-07-15

**Authors:** Chyntia Olivia Maurine Jasirwan

**Affiliations:** 1 Universitas Indonesia and Dr. Cipto Mangunkusumo Hospital, Jakarta, Indonesia

Dear Author,

 Thank you for your thorough and satisfactory responses to all the reviewers' comments. We appreciate your diligence and care in addressing each question and suggestion comprehensively.

 Your detailed explanations and revisions to the manuscript have significantly enhanced its clarity and quality. The additional data and insights have strengthened the overall argument and contributed to a better understanding of the study's implications.

 After reviewing the revised manuscript, we are pleased to conclude that it meets the necessary standards for getting approved for indexing.

 Best regards,

 Chyntia Olivia Maurine Jasirwan

Is the work clearly and accurately presented and does it cite the current literature?

Yes

If applicable, is the statistical analysis and its interpretation appropriate?

Yes

Are all the source data underlying the results available to ensure full reproducibility?

Partly

Is the study design appropriate and is the work technically sound?

Yes

Are the conclusions drawn adequately supported by the results?

Yes

Are sufficient details of methods and analysis provided to allow replication by others?

Yes

Reviewer Expertise:

gastroenterology and hepatology

I confirm that I have read this submission and believe that I have an appropriate level of expertise to confirm that it is of an acceptable scientific standard.

# Reviewer report: Reviewer response for version 1

**DOI:** 10.5256/f1000research.161310.r286137

**Published:** 2024-06-27

**Authors:** Chyntia Olivia Maurine Jasirwan

**Affiliations:** 1 Universitas Indonesia and Dr. Cipto Mangunkusumo Hospital, Jakarta, Indonesia

The manuscript investigates the use of Mac-2-binding protein glycosylation isomer (M2BPGi) as a diagnostic marker for liver fibrosis in chronic hepatitis B (CHB) patients. The study aims to determine the optimal cut-off values for M2BPGi and other non-invasive fibrosis markers (APRI, FIB-4, and AAR) and assess the diagnostic performance of these markers, both individually and in combination through a stepwise diagnostic analysis.

 This manuscript clearly presents objectives, methods, results, and conclusions, includes a comprehensive literature review that situates the study within the context of existing research, and accurately and appropriately cites relevant studies.

 Suggestions :

   - Abstract: Briefly mention the statistical methods used in the analysis.

   - Introduction: Add a few sentences highlighting the specific advantages of non-invasive methods over liver biopsies.

   - Discussion of Limitations: Provide a more detailed discussion of the study's limitations, such as the cross-sectional design, potential biases, and generalizability of the findings.

   - Future Directions: Suggest specific areas for future research, such as validating the findings in larger, diverse populations.

   - Analytical Code: Provide the exact scripts or commands used in SPSS and STATA for the analyses. This can be included as supplementary files.

   Quality Control Measures: Please mention any quality control measures or calibration procedures for the laboratory tests and liver elastography.

   - Supplementary Material: Include supplementary material detailing the step-by-step process of data processing and analysis.

   Confidence Intervals: Include confidence intervals for key statistics such as AUC values and cut-off points to provide a sense of the estimates' precision.

   - Validation: Discuss the potential for external validation of the findings in other populations or settings.

   - Subgroup Analysis: Perform and discuss subgroup analyses (e.g., based on age and gender) to provide additional insights.

   - Processed Data: Include processed data and the analytical code used for statistical analyses.

   - Detailed Data Files: Ensure data files are well-documented with clear labels and descriptions of each variable.

   - Reproducibility Checklist: Provide a checklist or protocol outlining all steps from data collection to final analysis.

   - Limitations: Expand on the limitations section to provide a balanced perspective.

   - Future Research Directions: Include specific suggestions for future research based on the study’s findings.

   - Confidence Intervals and Effect Sizes: Include these in the results to provide a clearer sense of the precision and reliability of the estimates.

 Conclusion

 The manuscript is scientifically sound, with a clear presentation, appropriate study design, and robust statistical analysis. Minor enhancements, such as providing detailed analytical code, discussing limitations more explicitly, and suggesting future research directions, will further strengthen the manuscript. Addressing these points will ensure the article meets high standards of scientific rigor and transparency.

Is the work clearly and accurately presented and does it cite the current literature?

Yes

If applicable, is the statistical analysis and its interpretation appropriate?

Yes

Are all the source data underlying the results available to ensure full reproducibility?

Partly

Is the study design appropriate and is the work technically sound?

Yes

Are the conclusions drawn adequately supported by the results?

Yes

Are sufficient details of methods and analysis provided to allow replication by others?

Yes

Reviewer Expertise:

gastroenterology and hepatology

I confirm that I have read this submission and believe that I have an appropriate level of expertise to confirm that it is of an acceptable scientific standard, however I have significant reservations, as outlined above.

# Response

**Published:** 2024-07-10

**Authors:** MUHAMMAD BEGAWAN BESTARI

**Affiliations:** Internal Medicine, Universitas Padjadjaran, Bandung, West Java, Indonesia

The F1000 Research

 Dear Editor and reviewer (Dr Chyntia Olivia Maurine Jasirwan),

 Subject: Submission of Revised Manuscript

 I hope this comment finds you well. I am writing to express my sincere gratitude for the feedback provided by the reviewer on our manuscript, “Revisiting Mac-2-Binding Protein Glycosylation Isomer (M2BPGi) for Diagnosing High-Risk Liver Fibrosis: A Stepwise Diagnostic Analysis.” The insightful comments and suggestions have significantly enhanced the quality and clarity of our work.

 In response to the reviewer's feedback, we have carefully addressed each comment and made substantial revisions to improve the overall quality of the manuscript. The critical revisions include:

 Limitations and Future Directions:

 We have added a discussion of potential limitations and future research directions from our research.

 Research steps, analysis, and results:

 We have provided additional files for research steps and analysis. The research results and discussion elaboration are presented more thoroughly.

 Supplementary Files:

 We have completed and added supplementary files from our paper to support reproducibility and understanding.

 Enclosed is the revised manuscript with tracked changes marked up the specific changes made in response to the reviewer's suggestions. These revisions address the reviewer's concerns and improve the manuscript's quality. We value the reviewer's time and effort and believe our manuscript now meets the high standards of F1000 Research.

 Thank you for considering our resubmission. We look forward to your response and the potential approval of our work for full publication.

 Kind Regards,

 Authors

**Suggestions from the reviewer:**

**1. Abstract: Briefly mention the statistical methods used in the analysis.**

**Author response:**

 Thanks for the suggestion.

 We have added the statistical methods used in the abstract section.

**2. Introduction: Add a few sentences highlighting the specific advantages of non-invasive methods over liver biopsies.**

**Author response:**

 Thanks for the suggestion.

 In the introduction section, We added a few sentences highlighting the advantages of non-invasive liver fibrosis assessment.

**3. Discussion of Limitations: Provide a more detailed discussion of the study's limitations, such as the cross-sectional design, potential biases, and generalizability of the findings.**

**Author response:**

 Thanks for the suggestion.

 In the discussion section, we have added a more detailed discussion regarding research limitations.

**4. Future Directions: Suggest specific areas for future research, such as validating the findings in larger, diverse populations.**

**Author response:**

 Thanks for the suggestion.

 We have completed the suggestions for future research in the last paragraph of the discussion section.

**5. Analytical Code: Provide the exact scripts or commands used in SPSS and STATA for the analyses. This can be included as supplementary files.**

**Author response:**

 Thanks for the suggestion.

 We have added a reproducibility checklist for the analytical code information to the extended data.

**6. Quality Control Measures: Please mention any quality control measures or calibration procedures for the laboratory tests and liver elastography.**

**Author response:**

 Thanks for the suggestion.

 In the methods section, We added information about the quality control and calibration for the laboratory tests and liver elastography.

**7. Supplementary Material: Include supplementary material detailing the step-by-step process of data processing and analysis.**

**Author response:**

 Thanks for the suggestion.

 We have added a reproducibility checklist to the extended data, detailing the step-by-step process of data processing and analysis.

**8. Confidence Intervals: Include confidence intervals for key statistics such as AUC values and cut-off points to provide a sense of the estimates' precision.**

**Author response:**

 Thanks for the suggestion.

 We have completed the confidence intervals and AUC data found and needed to interpret the research results as a whole.

**9.** **Validation: Discuss the potential for external validation of the findings in other populations or settings.**

**Author response:**

 Thanks for the suggestion.

 We have completed suggestions for validating our research results in the last paragraph of the discussion section.

**10.** **Subgroup Analysis: Perform and discuss subgroup analyses (e.g., based on age and gender) to provide additional insights.**

**Author response:**

 Thanks for the suggestion.

 We have added the results of the subgroup analysis in the discussion section to support and explain the overall research results.

**11. Processed Data: Include processed data and the analytical code used for statistical analyses.**

**Author response:**

 Thanks for the suggestion.

 We have completed the processed data in the Data Availability section and the analytical code can be seen in the reproducibility checklist in the extended data.

**12. Detailed Data Files: Ensure data files are well-documented with clear labels and descriptions of each variable.**

**Author response:**

 Thanks for the suggestion.

 We have completed the underlying data with clear labels and descriptions.

**13. Reproducibility Checklist: Provide a checklist or protocol outlining all steps from data collection to final analysis.**

**Author response:**

 Thanks for the suggestion.

 We have added a reproducibility checklist in the extended data for all steps from data collection to final analysis for the Stepwise Diagnostic Analysis.

**14. Limitations: Expand on the limitations section to provide a balanced perspective.**

**Author response:**

 Thanks for the suggestion.

 In the final part of the discussion, we have dissected the potential limitations of our study.

**15.** **Future Research Directions: Include specific suggestions for future research based on the study’s findings.**

**Author response:**

 Thanks for the suggestion.

 We have completed suggestions for future research directions in the last paragraph of the discussion section.

**16. Confidence Intervals and Effect Sizes: Include these in the results to provide a clearer sense of the precision and reliability of the estimates.**

**Author response:**

 Thanks for the suggestion.

 We ran a categorical predictor variables analysis to form some models that would also predict categorical variables (HR or LR group). Through those analyses, the effect of the model was assessed through Nagelkerke R ^2^, which we have added in the results section. In addition, we have included all important confidence interval data, including CIs for each AUC, to find the cut-off value in the discussion section.

# Reviewer report: Reviewer response for version 1

**DOI:** 10.5256/f1000research.161310.r273641

**Published:** 2024-06-14

**Authors:** Kemal Fariz Kalista

**Affiliations:** 1 Universitas Indonesia, Depok, West Java, Indonesia; 2 Internal Medicine, Cipto Mangunkusumo Hospital, Jakarta, Jakarta, Indonesia

I think this is very good work. This study comparing M2BPGi with elastography and many scoring system to assess liver fibrosis like APRI, FIB-4 and AAR, so i think this is a strength of this study, because not many studies comparing M2BPGi with multiple scoring system like this study. I consider to add “patient with chronic hepatitis B” in study title, because population in this study only include patient with hepatitis B. In discussion, I also consider to elaborate further regarding why APRI cut off for diagnosing liver fibrosis (0.311) is lower than many previous studies. It would be appreciate if author more explain why APRI was excluded from the model, because I think APRI is quite easy way to do in daily practice. Regarding study title “stepwise diagnosis analysis” I would suggest the author to provide how to apply study result to real life practice, eg: when considering patient for further fibrosis assessment with elastography or liver biopsy or if the M2BPGi result < 1.4 and FIB-4 > 0.7 what author suggestion in this kind of situation. Minor revision maybe it would be better if in table 2, in variable APRI, FIB-4, AAR and M2BPGi row, add note which one is lower or higher than cut off level.

 I think this study is approved with reservation, need to add further revision.

Is the work clearly and accurately presented and does it cite the current literature?

Yes

If applicable, is the statistical analysis and its interpretation appropriate?

Yes

Are all the source data underlying the results available to ensure full reproducibility?

Yes

Is the study design appropriate and is the work technically sound?

Yes

Are the conclusions drawn adequately supported by the results?

Partly

Are sufficient details of methods and analysis provided to allow replication by others?

Yes

Reviewer Expertise:

Hepatology

I confirm that I have read this submission and believe that I have an appropriate level of expertise to confirm that it is of an acceptable scientific standard, however I have significant reservations, as outlined above.

# Response

**Published:** 2024-07-10

**Authors:** MUHAMMAD BEGAWAN BESTARI

**Affiliations:** Internal Medicine, Universitas Padjadjaran, Bandung, West Java, Indonesia

The F1000 Research

 Dear Editor and reviewer (Dr Kemal Fariz Kalista),

 Subject: Submission of Revised Manuscript

 I hope this comment finds you well. I am writing to express my sincere gratitude for the feedback provided by the reviewer on our manuscript, “Revisiting Mac-2-Binding Protein Glycosylation Isomer (M2BPGi) for Diagnosing High-Risk Liver Fibrosis: A Stepwise Diagnostic Analysis.” The insightful comments and suggestions have significantly enhanced the quality and clarity of our work.

 In response to the reviewer's feedback, we have carefully addressed each comment and made substantial revisions to improve the overall quality of the manuscript. The critical revisions include:

 Clarification of Title:

 We have added information to the title to better represent our manuscript's contents.

 Clarification of Results and Discussion:

 We have provided additional structural discussion regarding the results and analysis related to APRI and added considerations regarding finding similar conditions in clinical practice. The results table has also undergone adjustments to make it more straightforward.

 Enclosed is the revised manuscript with tracked changes marked up the specific changes made in response to the reviewer's suggestions. These revisions address the reviewer's concerns and improve the manuscript's quality. We value the reviewer's time and effort and believe our manuscript now meets the high standards of F1000 Research.

 Thank you for considering our resubmission. We look forward to your response and the potential approval of our work for full publication.

 Kind Regards,

 Authors

**Suggestions from the reviewer:**

**1. Add “patient with chronic hepatitis B” in study title, because population in this study only include patient with hepatitis B.**

**Author response:**

 Thanks for the suggestion.

 We have added information about Chronic Hepatitis B patients in the study title.

**2. Elaborate further regarding why APRI cut off for diagnosing liver fibrosis (0.311) is lower than many previous studies**

**Author response:**

 Thanks for the suggestion.

 After reviewing and comparing various articles, we have added arguments related to the APRI cut-off value from our analysis to the discussion section.

**3. More explain why APRI was excluded from the model.**

**Author response:**

 Thanks for the suggestion.

 The strong correlation with FIB-4 and weaker overall performance than FIB-4, obtained from the prefix analysis, would make APRI insignificant if included in the statistical analysis.

**4. Regarding study title “stepwise diagnosis analysis” I would suggest the author to provide how to apply study result to real life practice**

**Author response:**

 Thanks for the suggestion.

 We have added the steps to follow when applying our study results in real-life practice. This information is available at the end of the discussion section.

**5. Minor revision maybe it would be better if in table 2, in variable APRI, FIB-4, AAR and M2BPGi row, add note which one is lower or higher than cut off level**

**Author response:**

 Thanks for the suggestion.

 We added information to Table 2 to make it more straightforward.
